# Motor Control Exercises and Their Design for Short-Term Pain Modulation in Patients with Pelvic Girdle Pain: A Narrative Review

**DOI:** 10.3390/healthcare13050572

**Published:** 2025-03-06

**Authors:** Mirko Zitti, Alessandro Mantia, Fabiola Garzonio, Graziano Raffaele, Lorenzo Storari, Rachele Paciotti, Fabio Fiorentino, Rebecca Andreutto, Filippo Maselli

**Affiliations:** 1 Department of Human Neurosciences, Sapienza University of Rome, 00185 Rome, Italy; alessandromantia97@gmail.com (A.M.); fabiolagarzonio@gmail.com (F.G.); graziano.raffaele997@gmail.com (G.R.); lorenzo.storari93@gmail.com (L.S.); rachele.paciotti@gmail.com (R.P.); fiorentinof02@gmail.com (F.F.); andreuttorebecca@gmail.com (R.A.); masellifilippo76@gmail.com (F.M.); 2 IRCCS Ospedale Policlinico San Martino, 16132 Genoa, Italy

**Keywords:** sacroiliac joint, motor control, short term pain, exercise, narrative review

## Abstract

**Background:** Pelvic girdle pain (PGP) is described in the literature as a subgroup of low back pain (LBP), characterized by pain localized between the posterior iliac crest and the gluteal fold, particularly near the sacroiliac joints. This condition can manifest in different forms non-specific PGP, occurring during pregnancy or postpartum (pregnancy-related PGP), which represents the most prevalent form and non-pregnancy-related PGP, resulting from mechanical alterations caused by trauma or microtrauma. Specific PGP, associated with identifiable causes such as fractures, infections, or arthritis. Over the years, research has focused on identifying the most effective approaches for managing this condition and addressing its associated biopsychosocial impairments. The aim of this narrative review is to determine the types of motor control exercises (MCEs) used to reduce short-term pain in patients with PGP and to assess whether these exercises are designed in accordance with the principles of motor control (MC) theories. **Methods**: A narrative review was conducted through searches in various medical and rehabilitation databases, including MEDLINE (via PubMed), PEDro, Scopus, and Web of Science. The inclusion criteria of the review encompassed case studies, case reports, editorials, original research articles, randomized controlled trials (RCTs), and systematic reviews (SRs). **Results**: Six articles met the eligibility criteria, comprising two SRs and four RCTs, all of which were included in the qualitative analysis. Among these, two studies examine MCEs for non-pregnancy-related PGP, while all the other studies focus on pregnancy-related PGP. The exercises described focused on lumbar–pelvic stabilization or deep spinal muscle activation. Among the six included studies, five did not report statistically significant changes in pain outcomes, while only one study demonstrated a statistically significant improvement. **Conclusions**: The analysis highlighted that the exercises currently employed are generally unspecific and not systematically structured according to the principles outlined in MC theories. The available evidence, combined with the incorrect design of these exercises, does not allow for definitive conclusions regarding the efficacy of MCEs in reducing short-term pain in patients with both pregnancy-related and non-pregnancy-related PGP.

## 1. Introduction

Pelvic girdle pain (PGP) is described in the literature as a subgroup of low back pain (LBP) and is typically defined as pain occurring between the posterior iliac crest and the gluteal fold, particularly in the region of the sacroiliac joints [1,2]. PGP can lead to disability in daily activities [2,3] and is classified into different types based on its underlying causes and characteristics: pregnancy-related that occurs in 23–65% of pregnant women, post-partum that occurs in 10–30% of women not pregnancy-related, or post-partum-related with a frequency of 20–30% in population [4,5]. There are also specific scenarios, due to the presence of rheumatologic (arthritis and sacroiliitis), orthopedic (fractures), immunological pathologies [6], or other severe pathologies like as tumors, cauda equina syndrome, etc. [7,8].

Various factors, including genetics, physical and pathoanatomical conditions, psychosocial influences, hormonal changes, and neurophysiological mechanisms, are potentially associated with PGP. This musculoskeletal disorder can be mediated both peripherally and centrally. In cases where PGP is mechanically induced, an inhibition or alteration of motor control (MC) and lumbopelvic muscle activity is often observed [6]. However, it remains unclear whether poor MC contributes to the development of pain or if pain itself leads to MC dysfunction [2,9].

Importantly, pain is the predominant symptom reported by individuals with PGP [1], often worsening with activities such as standing, walking for more than 30 min, sitting, or transitioning between postures [10]. Additionally, cognitive–behavioral impairments, including anxiety and kinesiophobia, have been documented in these individuals and are associated with greater levels of disability [6]. To address these challenges, various therapeutic interventions have been explored in the scientific literature, aiming to reduce pain over the short, medium, and long term [2].

In addition to these issues, MC impairments are commonly observed in individuals with PGP. The most frequently reported MC clinical impairments include delayed activation of the deep spinal, hip, and abdominal muscles (e.g., rectus femoris and external oblique), asymmetries in gait patterns—such as pelvic, thoracic, and lumbar rotational amplitudes in the transverse plane—and postural misalignments, including passive sway in standing posture [6,11]. However, these findings primarily focus on a biomechanical perspective rather than an ecological one, which would be more aligned with contemporary MC theories [12,13]. To address these dysfunctions, motor control exercises (MCEs) are recommended in the literature. However, there remains considerable uncertainty regarding the optimal dosage, including factors such as frequency, intensity, duration, and progression of the exercises [14].

MC is defined as the processing of information by the central nervous system to organize musculoskeletal system in order to control posture and to generate coordinated movements and actions [13]. MC arises from the interaction of several systems, including the sensory/perceptual, cognitive, and motor systems. A key role is played by motor learning, which is described as the process of finding a solution to a task through the interaction of the individual with the task itself and the environment [13].

Over time, several theories have been formulated to explain the functioning of MC; these include the Reflex Theory, Hierarchical Theory, Motor Programming Theory, Systems Theory, Dynamic Action Theory, Theory of Parallel Distributed Processing, Activity-Oriented Theory, and Ecological Theory [15] (Table 1). However, as Cano de la Cuerda et al. [15] point out, there is still no agreement on a single theory or model that fully explains MC.

All the theories described above were analyzed in this study by Levin et al. [12] which provides a new interpretation of these theories. Two theoretical approaches are identified: the direct approach and the indirect approach. In the direct approach, the central nervous system directly activates muscles via higher brain centers. In the indirect approach, force production results from the specification of neurophysiological parameters that are modifiable but remain independent [12]. In the direct framework, the mechanical approach and the internal model representation are grouped, while in the indirect framework, dynamic systems, and ecological approaches are grouped [12].

Based on the various existing theories, it is essential to design exercises that stimulate a wide range of cortical and ecological processes. Consequently, MCEs should be grounded in principles that promote meaningful and lasting changes [16], integrating the diverse aspects of the different MC theories. Furthermore, these exercises should align with the principles of motor learning to ensure their effectiveness [17].

The purpose of this narrative review is to provide an overview of the most-used MCEs for short-term pain reduction in PGP, to map them, and to lay the basis for reflecting on whether these exercises truly possess the characteristics to effectively stimulate MC.

There is a limited number of reviews on this topic [1,11,18]. Most previous reviews have focused primarily on the chronic phase rather than the acute phase and have assessed the effectiveness of MCEs without considering the qualitative aspects of exercise design [1,18]. In our view, these limitations highlight a significant gap in the literature that warrants further investigation. A narrative review, with its broad, cross-sectional, and qualitative approach, is particularly suited to explore this gap and provides a more comprehensive understanding of the current state of motor control exercise implementation in the short term for PGP.

## 2. Materials and Methods

The search was designed to focus on articles available in the literature from 1 January 2013 to 31 March 2024, across multiple medical and rehabilitation databases, including MEDLINE (via PubMed), PEDro, Scopus, and Web of Science. Specific search strategies for each database were employed (Tables S1 and S2).

The review followed the methodology described by Gasparyan et al. [19], specifically developed for narrative reviews. While narrative reviews allow for a more flexible style, some PRISMA checklist items [20] were followed to ensure greater rigor, except for the risk of bias assessment and protocol registration, which are not required for this type of review.

Designed with the PICOs model, the structured question framework aimed to facilitate and strengthen the search strategies. The review included all studies that focused on patients with pregnancy and non-pregnancy-related PGP or sacroiliac dysfunction. The intervention review consisted of all the treatments that included MCEs and was compared with conventional rehabilitation treatment as usual or no treatment (TAU). Studies that included interventions with MCEs or lumbopelvic stabilization exercises (a type of exercise designed to improve the stability and control of the muscles surrounding the lower back and pelvis) were included. Articles were excluded if they did not have a control group or if they did not include MCE interventions. Furthermore, studies that did not include patients with PGP or sacroiliac dysfunction were excluded.

The primary aim is to map the MCEs currently utilized for short-term pain reduction in PGP and to evaluate their effectiveness as intended in their current application. We included all types of articles written in English, such as case studies, case reports, editorials, original research articles, randomized controlled trials (RCTs) and systematic reviews (SRs), but we excluded studies involving children and adolescents.

After completing the search, Rayyan software (Qatar Computing Research Institute, Qatar) (https://www.rayyan.ai/, accessed on 4 March 2025) was used for duplicate removal and for screening titles and abstracts of eligible articles. The selection of studies was performed by two independent reviewers (A.M.; G.R.) according to the eligibility criteria. The reviewers independently screened records that were identified, based on title and abstract, using an inclusion/exclusion criteria template. Any disagreements were resolved through discussion to reach a consensus between the two review authors. If necessary, a third reviewer was consulted to make the final decision based on majority consensus (M.Z.). At the end of this process, the full text of the articles was obtained, and the same procedure was used for full text screening.

### Data Extracion

Two reviewers (A.M.; G.R.) independently extracted the data using a standardized form, capturing relevant information such as authors and year of publication, number and characteristics of participants, training, outcome measures, and conclusions drawn by the authors.

## 3. Results

**Figure 1:**
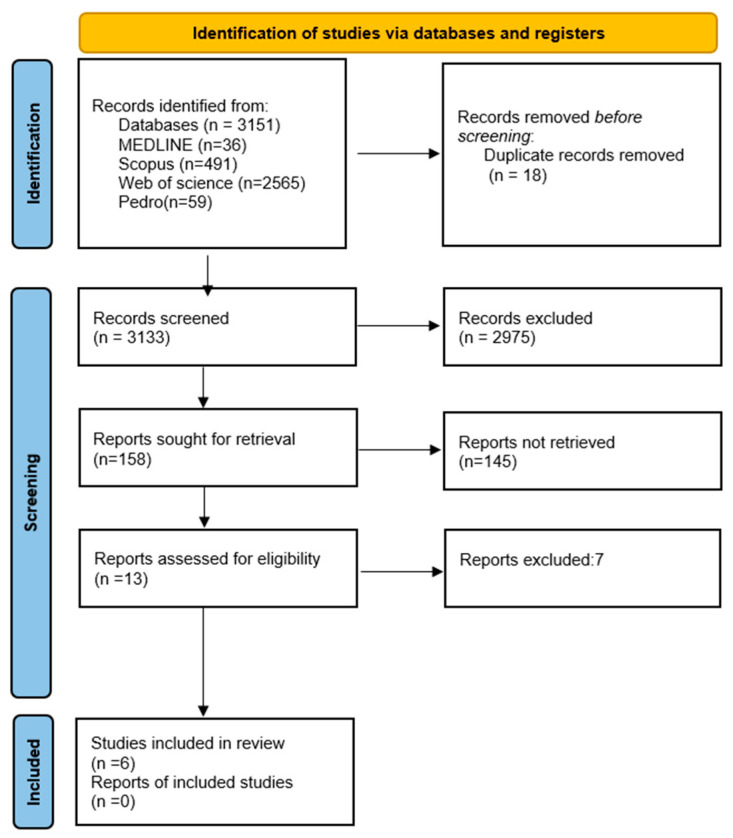
Flow chart of literature search [20].

The search strategy yielded a total of 3151 studies. Of the 3151 articles initially identified in the databases, 18 were removed due to duplication. Out of these, 13 were considered for inclusion after review of title and abstract. Following full-text screening only six articles met the eligibility criteria (Figure 1).

### Results of Studies Selected

The six studies included in this review were published between 2015 and 2023 and are summarized in Table 1. Four of these articles are RCTs, while two are SRs. Among these, two studies [21,22] examine MCEs for non-pregnancy-related PGP, while all the other studies [23,24,25,26] focus on pregnancy-related PGP. In all the included studies, the methods of exercise construction were analyzed to determine whether they aligned with MC theory (Table 2).

In the study by Kamali et al. [21], 30 patients with chronic to subacute PGP were included. Fifteen patients performed MCEs targeting pelvic tilt, strengthening pelvic muscles, and stabilizing muscles around the hip and pelvis, while the remaining fifteen patients received manipulation. No statistically significant differences were found between the MCE group and the control group in terms of pain and disability reduction.

The study by Nejati et al. [22] included 51 male and female patients who had experienced pain for at least three months. Participants were divided into three groups: the first group (19 participants) followed an exercise-only program that included stabilization exercises such as supine twists, bridging, quadruped exercises, and abdominal strengthening exercises. The second group (18 participants) received manual therapy, and the third group (19 participants) engaged in a combined exercise and manual therapy program. The exercise-only group showed a statistically significant improvement compared to the manual therapy-only group, but no significant difference was observed when compared to the combined exercise and manual therapy group.

Almousa et al. [23] analyzed 719 female patients with pregnancy-related PGP or PGP following childbirth. This review compared patients who performed stabilization exercises with those who did not. The study found no significant differences in pain or disability between the exercise and non-exercise groups.

In the study by Kokic et al. [24], 45 patients, all within the 30th week of pregnancy, were included. Twenty-three patients were assigned to the experimental group, and twenty-two to the control group. The experimental group performed aerobic and resistance exercises, while the control group received standard antenatal care. This study demonstrated a statistically significant improvement in the experimental group in terms of pain reduction and disability.

Abeer M. ElDeeb et al. [25] analyzed 40 patients with PGP during pregnancy or within the first three months postpartum. The patients were divided into two groups, each consisting of 20 participants: one intervention group and one control group. The intervention group performed exercises to improve pelvic floor muscle strength, while the control group engaged in lumbopelvic stabilization exercises. This study did not demonstrate significant improvements in the group performing isolated stabilization exercises compared to the group performing pelvic floor exercises.

In the study by Tseng et al. [26], four randomized controlled trials were included, involving 251 postnatal women either during pregnancy or within three months postpartum. The objective was to compare exercise programs specifically designed to strengthen deep local muscles and global muscles in the lumbopelvic region, with no therapy or physical therapy using other methods such as massage, relaxation, joint mobilization, manipulation, electrotherapy, hot packs, and simple back strengthening exercises. While there is some evidence indicating the effectiveness of exercise for relieving pain in pregnancy-related PGP, the most effective components of postnatal exercise programs for lumbopelvic pain treatment remain unclear. In this study, patients undergoing a supervised exercise program showed some benefits, but the results were not statistically significant in terms of pain reduction.

## 4. Discussion

The aim of our narrative review was to provide an overview of the most used MCEs for short-term pain reduction, mapping them, their characteristics and evaluating whether they truly align with MC principles.

This review synthesized findings from various study designs to outline the current state of the scientific literature regarding the design, implementation, and characteristics of MCEs for patients with PGP, both pregnancy-related and non-pregnancy-related. The primary objective was to assess whether these exercises, intended for short-term pain relief, are consistent with established MC theories.

To the authors’ knowledge, this is the first study in the literature to map the short-term structuring of MCEs for patients with PGP, which remains an under-researched condition, especially in rehabilitation. While pregnancy-related PGP has been extensively studied, scientific evidence on its non-pregnancy-related form remains limited [27]. Moreover, most research has focused on female populations, with a notable scarcity of studies involving male patients, a gap that is also reflected in our findings.

Notably, the analysis revealed a considerable degree of uniformity in the MCE described across studies [21,22,23,24,25,26].

These exercises were predominantly focused on stabilization or core stability, emphasizing muscle strengthening rather than inducing meaningful changes in MC [21,22,23,24,25,26]. This suggests a potential misunderstanding or misclassification of MCEs, where strengthening exercises are often conflated with true MC training.

Furthermore, the heterogeneity in exercise dosage and the variability in different forms of PGP, each with varying levels of symptom severity, may have influenced the short-term pain reduction outcomes. These inconsistencies make it challenging to determine the true effectiveness of MCEs in addressing PGP.

In terms of exercise structuring, while clinicians integrate various types of exercises, none appear to strictly adhere to MC theories, as described in the study by Cano de La Cuerda et al. [15]. This highlights the importance of clinical expertise and theoretical knowledge in designing effective MC interventions. A proper MCEs program should be based on a thorough understanding of MC principles rather than being reduced to muscle strengthening or stabilization exercises. Distinguishing between these concepts is crucial to ensuring that interventions are appropriately targeted and effective.

Key principles from MC theories, including attentional and learning aspects, as well as the top-down and bottom-up mechanisms regulating motor responses, have often been overlooked [13,16]. Bottom-up processing begins with peripheral sensory inputs (e.g., muscles, joints, and skin) and ascends through the central nervous system (e.g., spinal cord, brainstem, and motor cortex), playing a crucial role in reflexes and motor adaptation [13]. Conversely, top-down processing originates in higher brain areas (motor cortex and basal ganglia) and descends toward the spinal cord and muscles, regulating voluntary movements, motor planning, and movement inhibition [13]. The integration of these mechanisms is fundamental in exercise design, ensuring an optimal balance between sensory feedback and intentional MC [13,16].

In addition, none of the reviewed studies [21,22,23,25] incorporated essential elements such as external attentional focus, self-controlled practice, or positive feedback. These components are critical for stimulating MC and facilitating cortical and motor changes, as highlighted by Wulf G. et al. [16]. External focus, particularly, influences all stages of motor learning—acquisition, retention, and transfer. It involves directing attention not to the movement itself (internal focus) but to its effects on the surrounding environment [28,29]. This approach fosters more automated and efficient execution, leading to improvements in both performance and motor learning [28]. In the absence of these foundational features, it is difficult to claim that these exercises genuinely target MC improvement [14]. Modern motor control theories, such as the Activity-Oriented Theory and the Ecological Theory, emphasize the critical importance of dynamic interactions between the individual, the task, and the environment in shaping motor behavior [15]. The absence of this interaction in prescribed exercise programs constitutes a significant limitation, as it reduces the transferability of acquired skills to daily activities and compromises functional relevance [17].

For instance, incorporating real or contextually relevant objects can enrich the motor context, facilitating learning through realistic sensory stimuli and enhancing patient engagement. Integrating external focus cues, such as laser pointers to guide movement or mirrors for visual feedback, amplifies body awareness and promotes real-time motor adjustments. These approaches are supported by studies demonstrating that an external focus improves movement quality and efficiency compared to an exclusively internal focus [28,29].

Moreover, extrinsic feedback (e.g., vibrotactile cues or verbal instructions) can provide additional information to help patients discover optimal motor strategies, fostering exploration and adaptive learning. This aligns with Shumway-Cook’s models [13], which conceptualize motor control as an emergent process where perception and action are intrinsically linked to the environment.

The systematic integration of these elements into rehabilitation programs would not only enhance the ecological validity of therapeutic interventions but could also accelerate functional recovery, allowing patients to more effectively transfer skills learned in clinical settings to everyday life. Shifting towards a more contextually grounded, activity-oriented approach could represent a pivotal step in maximizing the efficacy of motor control interventions for pelvic girdle pain and other musculoskeletal conditions.

Another significant gap in the literature is the failure to address the acute phase of PGP, as none of the analyzed studies introduced MC exercises within the first four weeks post-symptom onset. This exclusion was based on findings suggesting that MC exercises are traditionally incorporated into rehabilitation programs only after this period. Furthermore, patient sample stratification was inconsistent. For example, in the study by Kamali et al. [21], the patient cohort ranged from acute/subacute (four weeks post-onset) to chronic (up to 11 years), yet no differentiation was made in rehabilitation plans. Similarly, Almousa et al. [23] included patients with PGP ranging from four weeks to one year in duration but did not individualize treatment accordingly. This lack of differentiation overlooked potential differences in peripheral and central sensitization between acute and chronic cases, potentially limiting the efficacy of the interventions.

However, emerging evidence challenges the rationale behind the delayed introduction of MC exercises [30]. Meier et al. [31] demonstrated that MC adaptations in acute LBP manifest early, characterized by alterations in lumbopelvic coordination, including changes in timing, magnitude, and kinematics. This suggests that maladaptive neuroplastic changes may begin in the initial phases of pain rather than emerging exclusively in chronic stages [31].

Since early neuromuscular adaptations, though initially protective, can contribute to pain chronicity if not addressed, delaying the implementation of MC exercises in PGP rehabilitation may represent a missed therapeutic opportunity [32]. Hodges et al. [32] highlighted that although early neuromuscular changes may serve a compensatory function, they can lead to long-term maladaptive reorganization of motor networks if not properly managed. Therefore, introducing targeted MC interventions within the first four weeks post-onset could mitigate these maladaptive changes, potentially improving long-term outcomes and reducing the risk of chronicity.

This approach aligns with contemporary neurophysiological models, which emphasize the role of early intervention in modulating central nervous system plasticity and optimizing movement strategies [12].

By reconsidering the timing of motor control exercise implementation, future research and clinical practice may enhance rehabilitation strategies for PGP, ultimately improving patient outcomes and minimizing the risk of persistent pain. While this aspect is more commonly emphasized in neurological rehabilitation [33], it is often underestimated in musculoskeletal rehabilitation.

These findings are consistent with those of Ganesh et al. [14], who questioned whether MCEs were appropriately designed to achieve their intended therapeutic goals. The similarities between their results and our observations further underscore the necessity of individualized, theory-driven approaches to MC rehabilitation in PGP.

### 4.1. Limits

This review has several limitations. First, the chosen study design: a narrative rather than a systematic review was selected to achieve the objective of mapping the topic. However, this approach carries inherent methodological limitations, such as the absence of a critical appraisal and a quantitative synthesis of the evidence. In summary, the common limitations observed across the studies include small sample sizes, the lack of an untreated control group, short follow-up durations, and the absence of participant blinding [21,22,23,25]. These methodological issues can potentially impact both the internal and external validity of the findings. Small sample sizes may lead to underpowered studies, reducing the reliability of the results and the ability to detect significant effects. The absence of an untreated control group makes it challenging to isolate the specific effects of the intervention, as it becomes difficult to account for the natural course of the condition or any placebo effects. Additionally, the short follow-up durations limit the ability to assess the long-term effectiveness and sustainability of the interventions. Finally, the lack of participant blinding introduces the potential for bias, as participants’ expectations or knowledge of the treatment may influence their responses. Collectively, these limitations underline the need for further research employing more robust study designs, including larger sample sizes, appropriate control groups, longer follow-up periods, and blinding to better confirm the results and provide stronger evidence for clinical practice.

Second, the search was restricted to the past 10 years. While this allowed for the inclusion and analysis of the most recent and relevant evidence, it limited the ability to provide a comprehensive overview of the condition and its rehabilitative approaches in earlier years. Additionally, the search did not include gray literature, which may have excluded relevant studies or perspectives.

Another limitation is the focus on female populations in the reviewed studies, reflecting a broader gap in the literature on non-pregnancy-related PGP, which could include male populations. This lack of research contributes to an incomplete understanding of the condition in non-pregnancy contexts. Moreover, the studies exhibited heterogeneity in the timing of the starting of MCEs, with no consensus on the optimal time to begin rehabilitation. This variability complicates the interpretation of results and their application to clinical practice.

### 4.2. Clinical Implication and Future Study Directions

In the future, addressing the significant gap in the literature should begin with establishing a more precise and universally shared definition of MC [34]. This calls for research aimed at clarifying and expanding our understanding of this concept. Further studies are essential to explore the structuring of MCEs within the field of rehabilitation, enabling the design and evaluation of interventions that focus exclusively on MCEs for managing PGP, particularly with respect to short-term pain reduction. Additionally, it is crucial to encourage clinicians to tailor exercises to individual patients, ensuring an optimal balance between the insights from various theoretical frameworks, principles of motor learning, and the specific needs of each patient. Specifically, this means designing personalized treatments based on the impairments identified in each patient and structuring exercises accordingly, referring to the principles of MC theory most appropriate to the specific impairment. This approach would not only enhance treatment effectiveness but also improve evaluation processes, allowing for a more detailed investigation of MC. Future pilot studies should be conducted to evaluate the feasibility and potential clinical benefits of this approach.

## 5. Conclusions

This analysis revealed that the exercises currently utilized are poorly structured and not specific enough, often failing to adhere to the principles proposed by various MC theories.

Consequently, the data currently available and the way these exercises are structured do not allow us to clearly determine whether MCEs are effective in reducing short-term pain in patients with pregnancy-related or non-pregnancy-related PGP, since they cannot truly be classified as MCEs.

In conclusion, future research is essential to enhance the understanding of MCE structuring within the field of rehabilitation; this will be crucial for designing and evaluating the effects of MCE approaches in managing PGP and their potential for short-term pain reduction.

## Figures and Tables

**Table 1:** Summary of motor control theory [15].

Motor Control Theory	Clinical Rationale
Reflex Theory 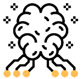	Motor control recovery adjusts reflex effects during tasks. The Bobath concept is partially based on this theory.
Hierarchical theory 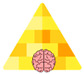	Reflex analysis helps assess motor control in neurological patients, gauge neural maturity, and predict function.
Motor programming theory 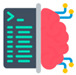	Focuses on relearning movement patterns, restoring key functional actions, and using alternative effectors if needed.
Systems theory 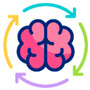	Assessment and treatment should address both specific motor control deficits and broader system interactions, including musculoskeletal deficiencies.
Dynamic action theory 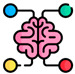	Motor behavior changes follow physical principles rather than just neural structures, guiding therapy through body dynamics.
Theory of parallel distributed processing 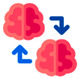	Brain systems are redundant, allowing function despite damage. This model helps predict how nervous system lesions affect performance.
Activity-oriented theory 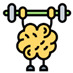	It states that MC recovery should focus on essential functional activities.
Ecological theory 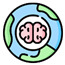	Describes the subject as an active explorer of their environment, where exploration enables the development of multiple ways to perform a task.

Note: the images provide a visual summary of the key concepts of the theories, while the “Clinical Rationale” represents a summarized description of the underlying mechanisms for each theory.

**Table 2:** Summary of studies included.

Author (Year)	Study Design	Group Characteristics	Rehabilitation Intervention	Outcome Measures	Conclusions
Almousa et al. (2017) [23]	SR	719 female individuals with PGP pregnancy related and post-partum.	IG: exercises for activation of the multifidus and transversus abdominis. Performed 3 times a week for 12 weeks. CG: no exercises.	Pain and disability.	There is limited evidence for the clinician to conclude on the effectiveness of stabilizing exercises in treating PGP during pregnancy and the postpartum periods.
ElDeeb et al. (2019) [25]	RCT	40 female individuals with PGP during pregnancy or within the first 3 months postpartum. IG: n = 20 patients. CG: n = 20 patients.	IG: exercises to activate the multifidus and transversus abdominis. Performed 3 times a week for 12 weeks. CG: exercises for strengthening pelvic floor muscle. Performed 3 times a week for 12 weeks.	Pain.	This study did not demonstrate significant improvements in the group performing isolated stabilization exercises compared to the group performing pelvic floor exercises.
Kamali et al.(2019) [21]	RCT	30 individuals with subacute or chronic sacroiliac joint dysfunction. IG: n = 10 F patients, 5 M. patients. CG: n = 11 F patients, 4 M patients	IG: stabilization exercise (control of neutral spine alignment, co-contraction of the local muscles transverse abdominis and deep fibers of multifidus with normal, etc.). Patients performed for 20 min 3 times a week, for 4 weeks. CG: in each session, the therapist performed a manipulation technique on the side with positive SIJ test results. Patients were treated individually 3 times a week for 2 weeks by a physiotherapist expert in manual therapy.	Pain and disability.	Despite the improvements seen after both manipulation and stabilization exercise therapies in patients with sacroiliac joint dysfunction, there was no significant between-group difference in the treatment effects.
Nejati et al.(2018) [22]	RCT	51 individuals (sex not specified) with lower back or buttock pain. EG: n = 19 MTG: n = 18 EMTG: n = 19	All groups performed exercises at home daily for 12 weeks. EG: group received posterior innominate self-mobilization, sacroiliac joint stretching, and spinal stabilization exercises. MG: spinal stabilization exercises EMTG: manual therapy plus stretch and self-mobilization exercises.	Pain and disability	Exercise only and manipulation plus exercise therapy appear to be effective in reducing pain and disability in patients with sacroiliac joint dysfunction.
Kokic et al. (2017) [24]	RCT	45 female individuals with PGP by the 30th week of pregnancy.	IG: aerobic activity and resistance exercises. Activation of the lumbopelvic musculature, pelvic floor exercises, and final stretching. The exercise program commenced within 1 week following inclusion into the trial and continued throughout the duration of the pregnancy. CG: received only standard antenatalcare, but were not discouraged from exercising on their own.	Pain and disability	The exercise program had a beneficial effect on the severity of lumbopelvic pain in pregnancy, reducing the intensity of pain and the level of disability experienced as a result.
Tseng et al. (2015) [26]	SR	251 female individuals with PGP during pregnancy or within the first three months postpartum.	IG: stabilization exercises for the lumbopelvic muscles specifically the multifidus, abdominal muscles, and transversus abdominis. CG: manual therapy, mobilizations, manipulation, and massage.	Pain	There is some evidence to indicate the effectiveness of exercise for relieving lumbopelvic pain, further good quality trials are needed to ascertain the most effective elements of postnatal exercise programs suited for LPP treatment.

Abbreviations list: RCT = randomized controlled trial; SR = systematic review; M = male; F = female; CG = control group; IG = intervention group; MCE = motor control exercise; PGP = pelvic girdle pain; EG = exercise group; MTG = manual therapy group; EMTG = exercise manual therapy group; SIJ = Sacro Iliac joint.

## Data Availability

Data are available upon reasonable request to the corresponding author.

## Supplementary Materials

The following supporting information can be downloaded at: https://www.mdpi.com/article/10.3390/healthcare13050572/s1, Table S1. Search strategy across multiple databases. Table S2. Research limitations.
