# Supplementary material for: Motor Control Exercises and Their Design for Short-Term Pain Modulation in Patients with Pelvic Girdle Pain: A Narrative Review
Source: Healthcare (Basel). 2025 Mar 6;13(5):572. doi: 10.3390/healthcare13050572 (PMC11899138; doi:10.3390/healthcare13050572)
Supplement: Supplementary file 1 [file healthcare-13-00572-s001.zip › healthcare-3458701-supplementary.pdf]

## Supplementary Materials

**Table S1.** Search strategy across multiple databases.

| |
|---|
| *PubMed research string*: ((((('pelvic girdle pain'[MeSH Terms]) OR ('sacroiliac joint pain'[MeSH Terms])) ) OR (PGP)) AND ((('motor control exercise') OR (exercise[MeSH Terms])) OR ('stabilising exercise'))) AND ((((('short term outcome') OR (pain)) OR (rehabilitation))) |
| *Web Of Science research string :* (((((ALL=('Pelvic girdle pain')) OR ALL=(PGP)) OR ALL=('Sacroiliac joint pain')) AND ALL=('motor control exercise')) OR ALL=('stabilising exercise')) |
| *PEDro free terms:* Pelvic girdle pain; Sacroiliac joint pain |
| *Scopus research string:*( ( TITLE-ABS-KEY ( pelvic AND girdle AND pain ) OR TITLE-ABS-KEY ( sacroiliac AND joint AND pain ) OR TITLE-ABS-KEY ( pgp ) ) ) AND ( ( TITLE-ABS-KEY ( motor AND control AND exercise ) OR TITLE-ABS-KEY ( stabilising AND exercise ) OR TITLE-ABS-KEY ( exercise ) ) ) AND ( ( TITLE-ABS-KEY ( short AND term AND outcome ) OR TITLE-ABS-KEY ( pain ) OR TITLE-ABS-KEY ( physiotherapy ) ) ) |

**Table S2.** Research limitations

| **Limitations** | | |
|---|---|---|
| **Language** | **Time Frame** | **Research method's** |
| Only English sources were considered. | The research focused on publications from January 1, 2013, to March 31, 2024. | No grey literature was investigated. |
